# Development of a Physiologically Based Pharmacokinetic Model for Tegoprazan: Application for the Prediction of Drug–Drug Interactions with CYP3A4 Perpetrators

**DOI:** 10.3390/pharmaceutics15010182

**Published:** 2023-01-04

**Authors:** Lien Thi Ngo, Jaeyeon Lee, Hwi-yeol Yun, Jung-woo Chae

**Affiliations:** College of Pharmacy, Chungnam National University, Daejeon 34134, Republic of Korea

**Keywords:** tegoprazan, PBPK model, drug–drug interaction, CYP3A4, metabolism

## Abstract

Tegoprazan is a novel potassium-competitive acid blocker (P-CAB) developed by CJ Healthcare (Korea) for the treatment of gastroesophageal reflux disease and helicobacter pylori infections. Tegoprazan is mainly metabolized by cytochrome P450 (CYP) 3A4. Considering the therapeutic indications, tegoprazan is likely to be administered in combination with various drugs. Therefore, the investigation of drug–drug interactions (DDI) between tegoprazan and CYP3A4 perpetrators is imperative. In the present study, we first aimed to develop a physiologically based pharmacokinetic (PK) model for tegoprazan and its major metabolite, M1, using PK-Sim^®^. This model was applied to predict the DDI between tegoprazan and CYP3A4 perpetrators. Clarithromycin, a potent inhibitor of CYP3A4, and rifampicin, a strong inducer of CYP3A4, were selected as case studies. Our results show that clarithromycin significantly increased the exposure of tegoprazan. The area under the concentration–time curve (*AUC*) and *C*_max_ of tegoprazan in the steady state increased up to 4.54- and 2.05-fold, respectively, when tegoprazan (50 mg, twice daily) was coadministered with clarithromycin (500 mg, three times daily). Rifampicin significantly reduced the exposure of tegoprazan. The *AUC* and *C*_max_ of tegoprazan were reduced by 5.71- and 3.51-fold when tegoprazan was coadministered with rifampicin (600 mg, once daily). Due to the high DDI potential, the comedication of tegoprazan with CYP3A4 perpetrators should be controlled. The dosage adjustment for each individual is suggested.

## 1. Introduction

Tegoprazan, a novel, potassium-competitive acid blocker (P-CAB) developed by CJ Healthcare (South Korea), was approved in South Korea for the treatment of gastroesophageal reflux disease (GERD) in 2019 and for the treatment of gastric ulcers and *Helicobacter pylori* infections in 2020 [1,2]. Tegoprazan exhibits its antisecretory effects by competitively and reversibly blocking the availability of K+ of the H+, K+-ATPase [2,3].

The major metabolic pathway of tegoprazan is in the liver, and the kidney excretes a negligible amount [2,3]. Both in vitro and clinical results have elucidated that tegoprazan is a potential substrate of cytochrome P450 (CYP) 3A4 [2,3,4,5,6]. An in vitro study reported that ketoconazole, a strong inhibitor of CYP3A4, significantly inhibited the metabolism of tegoprazan in human liver microsomes [5]. When tegoprazan was coadministered with another potent inhibitor of CYP3A34 (clarithromycin) in a clinical study, the maximum concentration (*C*_max_) and area under the concentration curve (*AUC*) during one dosing interval in the steady state of tegoprazan were increased 2.2-fold and 2.7-fold, respectively, compared with the administration of tegoprazan alone [6]. In addition to CYP3A4, it has been reported that tegoprazan is metabolized partly by CYP2C19 [2,3,4]. In the presence of recombinant CYP3A4 and CYP2C19, the intrinsic clearance of tegoprazan was 0.86 and 0.61 µL/min/pmol protein, respectively [4]. The metabolite M1 (desmethyl tegoprazan) is the major metabolite of tegoprazan, which equals 6.8% of the total administered tegoprazan dose. M1 is formed by both the metabolic reaction of CYP3A4 and CYP2C19. The metabolic rate of M1 and tegoprazan is approximately 1.44–1.52 [3]. Compared with tegoprazan, M1 showed 10-fold less potency in its inhibitory potential against porcine H+/K+-ATPase, with a half-maximal inhibitory concentration value of 6.19 µM compared with 0.53 µM for the parent drug [1,7].

Considering the therapeutic indications, tegoprazan is likely to be administered in combination with various drugs. Therefore, the investigation of drug–drug interactions (DDI) between tegoprazan and other CYP3A4 perpetrators is imperative. Commonly, DDI profiles are investigated in clinical trial studies. However, conducting clinical trials is costly and time-consuming; in addition, there might be unknown potential risks to the participants. Therefore, predictions based on PK model simulations using in vitro and in vivo data have received a lot of attention due to their many advantages in the prediction of DDIs [8,9,10].

In the literature, two physiologically based pharmacokinetic (PBPK) models for tegoprazan have been reported, developed by Yoon et al. [5] and Jeong et al. [4]. Both studies used the commercially available software Simcyp Simulator to build the PBPK model and generate the PK simulations. In the study by Yoon et al. [5], a PBPK model for tegoprazan was developed using several clinical pharmacokinetic (PK) profiles of tegoprazan following the administration of a single dose (25, 50, and 100 mg) or multiple doses (50 and 100 mg). However, the PBPK model for the metabolite M1 and the effect of food on the PK of tegoprazan (and, consequently, the PK of its metabolite) was not considered [5]. In the study by Jeong et al. [4], an improved PBPK model for tegoprazan and M1 to overcome the limitation of the previous model was reported. However, in this study, the plasma concentration–time profiles of tegoprazan and M1 from only one clinical study (subjects received a single dose of tegoprazan 100mg) were used for the model’s development. Meanwhile, many PK profiles of tegoprazan and M1 are available in the literature.

The objective of this study was to develop a PBPK model for both tegoprazan and M1 using a free-to-everyone modeling and simulation tool, PK-Sim^®^ (Open Systems Pharmacology Suite 9.1, licensed from the Open Systems Pharmacology community www.open-systems-pharmacology.org, accessed on 02 February 2022) considering that the library of PBPK models using PK-Sim^®^ has been added to day by day. The model’s development used all the latest clinical PK profiles of tegoprazan and M1. After the evaluation, the model was applied to predict the DDI profiles of tegoprazan with CYP34 perpetrators. Clarithromycin (a strong CYP3A4 inhibitor) and rifampicin (a strong CYP3A4 inducer) were selected as two case studies to assess the model’s performance in the prediction of the DDI profiles of tegoprazan.

## 2. Materials and Methods

### 2.1. PBPK Model Development and Evaluation

The PBPK model of tegoprazan and tegoprazan metabolite, M1, was developed using the PK-Sim^®^ software. The clinical PK data of tegoprazan and M1 used for model development (training datasets) and evaluation (test datasets) were extracted from previous publications using the WebPlotDigitizer tool (https://automeris.io/WebPlotDigitizer/, accessed on 15 February 2022) [11]. Publications about tegoprazan and M1 were searched in the National Library of Medicine (https://pubmed.ncbi.nlm.nih.gov/, accessed on 10 February 2022) with the keywords “tegoprazan” and “CJ-12420”. All the available clinical PK data were included in this study without any exclusion criteria. In summary, a total of 19 clinical studies (10 training and 9 test datasets; Table S1, Supplementary Materials) with the plasma concentration–time profiles of tegoprazan and M1 after the oral administration of tegoprazan in humans were used. These studies covered an extensive dose range (from 50 to 400 mg) after a single dose or repeated doses of tegoprazan in both the fasted and fed states.

The PBPK model was used on virtual individuals for model development and evaluation. The demographics (i.e., race, sex, age, weight, and body-mass index) were generated to simulate individuals in clinical studies. All human physiological parameters were provided within the software. The relative tissue expressions of enzymes and transporters were extracted from reverse transcription polymerase chain reaction (RT-PCR) profiles from the PK-Sim^®^ database whenever available. Drug-dependent parameters (belonging to “compounds” building blocks) and formulation-dependent parameters (belonging to “formulations” building blocks) were extracted from the literature if available. Parameters that were not available from the literature were optimized by fitting the model to the observed training dataset. Finally, some model parameters were refined to obtain the best fit.

In detail, the PBPK model for tegoprazan was first developed using the whole PK profiles of tegoprazan in the training dataset. The hepatic clearance of tegoprazan by CYP3A4 and by CYP2C19, regardless of metabolic products, and the renal clearance of tegoprazan were included. The metabolic enzyme activity rate was described as a first-order process in the presence of recombinant CYPs (rCYPs), as described in the equation below.

(1) $$v_{CYP}=\left[E\right]*CL_{vitro,CYP}$$

where [*E*] is the *CYP* concentration (pmol), and $CL_{vitro,CYP}\;$ is the in vitro clearance (µL/min/pmol protein) of the compound in the presence of the *CYP*. The elimination of tegoprazan in the kidney is described through a parameter of total plasma clearance, as presented in the equation below.

(2) $$v_{renal}=CL_{plasmac,\;renal}$$

where $CL_{plasma,\;renal}\;$ is the total plasma clearance (mL/min/kg body weight) of tegoprazan through the kidneys. The initial value for the metabolic reaction was picked up from a published in vitro study [4] after correction using the intersystem extrapolation factors (ISEFs) that integrate the variables of intrinsic activity and accessory protein expression between two systems (rCYPs and human liver microsomes) [12,13]. This study used ISEFs of 0.21 and 0.25 for CYP3A4 and CYP2C19, respectively.

A Weibull function was selected to describe the dissolution profile of tegoprazan from the drug formulation due to its ability to fit almost any kind of dissolution curve. Accordingly, the accumulated fraction of the drug (m) in solution at time *t* can be described following the equation below [14,15].

(3) $$m=1-\exp\left(\frac{-{\left(t\right)}^{b}}{a}\right)$$

where “*a*” is the scale parameter of the Weibull function, corresponding to the dissolution time, defining the time after the start of dissolution as being when 50% of the administered dose is dissolved. “*b*” is the shape parameter of the Weibull function, corresponding to the dissolution shape.

For model development, the metabolic enzyme activities, lipophilicity, specific intestinal permeability, and dissolution profiles under the fasted and fed states were simultaneously estimated. The estimated parameters derived from the PBPK model of tegoprazan were then applied as the initial parameters to develop the PBPK model for tegoprazan and the metabolite M1.

The PBPK model of M1 was built using hepatic clearance, which was described as a first-order process in the liver. The hepatic elimination of M1 was described through a parameter of total hepatic plasma clearance, as presented in the equation below.

(4) $$v_{hepatic}=CL_{plasmac,\;hepatic}$$

In this step, the metabolism reaction of tegoprazan was modeled in consideration of the metabolism products. In detail, the following enzyme activities were included: (1) the CYP3A4 reaction to form M1 metabolite, (2) the CYP3A4 reaction to form metabolites other than M1, (3) the CYP2C19 reaction to form M1 metabolite, and (4) the CYP2C19 reaction to form metabolites other than M1. The rate of the metabolic enzyme activity was described following Equation (1). The parameters of lipophilicity, specific intestinal permeability, metabolism–reaction activities, and formulation–dissolution profiles were finally optimized by fitting the model to the whole PK profiles of tegoprazan and M1 in the training dataset. Of note, the PK profile of M1 in the study, when tegoprazan was coadministered with clarithromycin (ID 09), was not included in the dataset used for model development. A summary of clinical datasets and parameters used in the PBPK model are listed in Table 1 and Tables S1 and S2 (Supplementary Materials).

To assess the performance of a PBPK model during model development and evaluation, the predicted and observed values of (1) the plasma concentration–time profiles and (2) the PK parameters of the drug were compared. In addition, the mean relative deviation (*MRD*) of the predicted plasma concentrations and the geometric mean fold error (*GMFE*) of the predicted PK parameters of the drug were calculated. The *MRD* and *GMFE* were calculated as described in the equations below.

(5) $$MRD=10^{x},\;\mathrm{with}\;x=\sqrt{\frac{1}{m}\sum_{i=1}^{m}{\left(log_{10}C_{pred,i}-log_{10}C_{obs,i}\right)}^{2}\;}$$

(6) $$GMFE=10^{x},\;\mathrm{with}\;x=\frac{1}{n}\sum_{i=1}^{n}\left|\left(log_{10}PK_{pred,i}-log_{10}PK_{obs,i}\right)\right|$$

where $C_{pred,i}$ and $C_{obs,i}$ are the predicted and observed plasma concentrations, respectively. m is the number of observed concentrations.

### 2.2. Sensitivity Analysis

A sensitivity analysis was performed to assess which input parameters majorly affected the PK profile of the compound. Parameters that were optimized, associated with optimized parameters, or significantly variated due to calculation methods were included in the analysis. For tegoprazan, the tested parameters included enzymatic reactions, specific intestinal permeability, dissolution time and shape, solubility, lipophilicity, and the fraction unbound in plasma. For M1, the tested parameters included the total hepatic clearance and lipophilicity of M1.

### 2.3. Prediction DDI Profile of Tegoprazan

After evaluating the tegoprazan PBPK model, an integrated PBPK model of tegoprazan with clarithromycin and rifampicin was used to simulate the DDI profile of tegoprazan. The simulation was carried out in virtual populations (*n* = 100, 100% male) with an age range of 19 to 45 years, a body-mass index ranging from 19.00 to 28.00, and a weight of 50kg. The PBPK models of clarithromycin and rifampicin were derived from the OSP-PBPK-Model-Library (https://github.com/Open-Systems-Pharmacology/OSP-PBPK-Model-Library, accessed on 25 April 2022) [19,20,21,22,23,24]. For tegoprazan, two dosage scenarios were simulated, including 50 mg once or twice daily. In cases of DDI studies, the oral administration of 600 mg of rifampicin once daily and 500 mg of clarithromycin twice or three times daily were simulated. These dosage scenarios were set up based on their clinical dosages.

The CYP3A4 inhibition effect of clarithromycin was described using an inactivator irreversibly inhibiting model (mechanism-based inactivation model) [25]. The induction effect of rifampicin was described using the increase in the de novo synthesis of the target via an Emax model.

(7) $$R_{syn,ind}=R_{syn}\times \left(1+\frac{E_{max}\times \left[Ind\right]}{EC_{50}+\left[Ind\right]}\right)$$

where $R_{syn}$ and $R_{syn,ind}$ are the synthesis rates of the enzyme in the absence and presence of the inducer rifampicin, respectively; *E_max_* is the maximal induction effect of rifampicin; *EC*_50_ is the concentration of rifampicin that causes a half-maximal induction effect; and [*Ind*] is the rifampicin concentration.

## 3. Results

### 3.1. PBPK Model Development and Evaluation

Whole-body PBPK models of tegoprazan and M1 were developed and evaluated using a total of 19 clinical studies (10 training datasets and 9 test datasets; Tables S1 and S2, Supplementary Materials; PK-Sim Model File, Supplementary Materials).

In summary, the PBPK model of tegoprazan was first developed using the whole PK profiles of tegoprazan in the training datasets. The clearance of tegoprazan by CYP3A4 and CYP2C19 was estimated regardless of metabolic products. The initial value of the metabolic reaction was derived from a published in vitro study [4] after correction using the ISEFs that integrate the variables of intrinsic activity and accessory protein expression between rCYPs and human liver microsomes [12,13]. Consequently, the intrinsic clearance rates of tegoprazan by CYP3A4 and CYP2C19 were estimated as 0.247 and 0.340 μL/min/pmol protein, respectively. The estimated parameters derived from the PBPK model of tegoprazan (including metabolic reaction rates, intestinal permeability, lipophilicity, and the dissolution profile of tegoprazan under the fasted and fed states) were then applied as the initial parameters to develop the PBPK model for tegoprazan and the metabolite M1.

The final model parameters of the PBPK model of tegoprazan and M1 are listed in Table 1. In this step, the metabolism reaction of tegoprazan was modeled in consideration of the metabolism products. The result was that CYP3A4 metabolized tegoprazan to form M1 and metabolites other than M1 at a rate of 9.29 × 10^−3^ and 0.236 μL/min/pmol protein, respectively. With CYP3A4, the metabolite M1 was equal to 2.71%, and other metabolites were equal to 68.9% of the administered tegoprazan dose. Respectively, CYP2C19 metabolized tegoprazan to form M1 and metabolites other than M1 at a rate of 0.0921 and 0.242 μL/min/pmol protein. With CYP2C19, the metabolite M1 and metabolites other than M1 were equal to 5.57% and 14.6% of the administered tegoprazan dose, respectively. The renal plasma-clearance parameter modeled the excretion of tegoprazan via the kidneys. A value of 0.297 mL/min/kg (1.31 L/h) was identified from the published data [18]. The metabolite M1 was transformed from its parent compound, tegoprazan, via both CYP3A4 and CYP2C19. In addition, the following parameters were included in the PBPK model of M1: molecular weight, fraction unbound in plasma, and pKa were identified from the available data. The solubility was fixed at a default value of 1 ng/mL. The lipophilicity, which was assumed to be the same as tegoprazan, and the total hepatic clearance were estimated using the parameter identification function.

**Figure 1:**
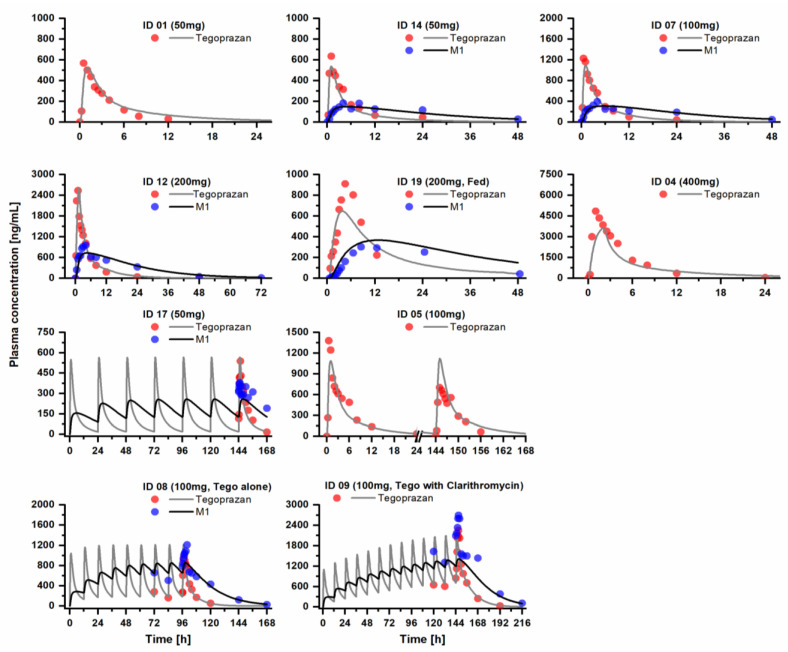
In terms of the training dataset, the predicted and observed plasma concentration–time curves of tegoprazan and the metabolite M1 after the oral administration of tegoprazan in healthy subjects are shown. Solid lines are the predicted values of each model. Circles are clinical observations. Details on dosing regimens, characteristics, subject demographics, and references are listed in Table S1. The PK profile of M1 in the ID 09 was not used for model development. The simulation for M1 in this ID was performed based on the developed PBPK models of tegoprazan and M1.

**Figure 2:**
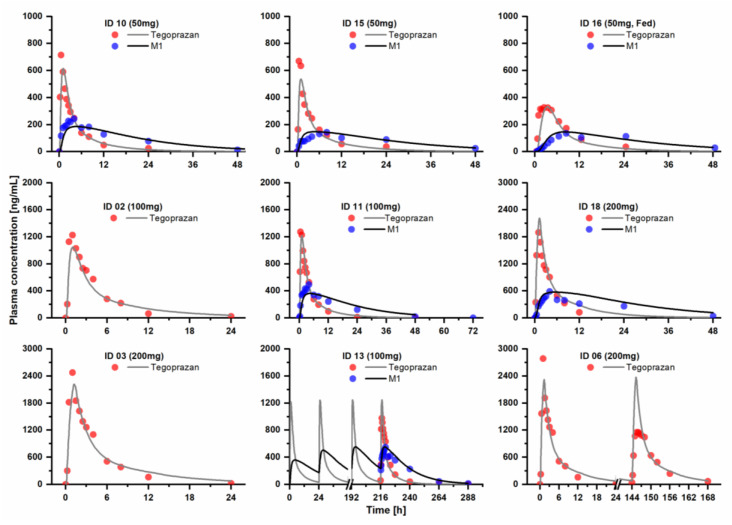
In terms of the test dataset, the predicted and observed plasma concentration–time curves of tegoprazan and the metabolite M1 after the oral administration of tegoprazan in healthy subjects are shown. Solid lines are the predicted values of each model. Circles are clinical observations. Details on dosing regimens, characteristics, subject demographics, and references are listed in Table S1.

**Figure 3:**
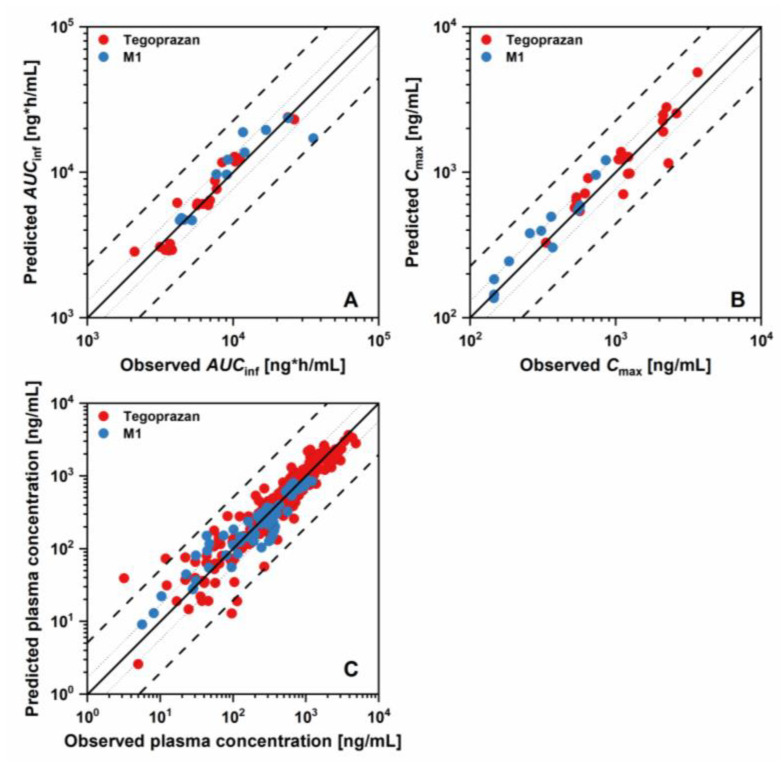
Goodness-of-fit plots for the developed PBPK model of tegoprazan for the prediction of (**A**) plasma concentration, (**B**) Cmax, and (**C**) *AUC*_inf_. The line of identity is presented as a solid line; 1.25-fold dimensions and 2.0-fold dimensions are shown using dotted lines and dashed lines, respectively.

The results of the evaluation steps for the developed models are presented in Figure 1, Figure 2 and Figure 3 and Tables S1 and S2 (Supplementary Materials), showing that the developed model described the PK profiles of tegoprazan and its metabolite, M1, effectively. The GMFE values for the *AUC*_inf_ of tegoprazan and M1 were 1.158 and 1.200, respectively. The GMFE values for the *C*_max_ of the tegoprazan and M1 were 1.214 and 1.201. Moreover, 90.6% and 89.7% of all the predicted plasma concentrations for the tegoprazan and M1 fell within the two-fold dimensions of the corresponding observed concentrations. The overall MRD values for the predicted plasma concentrations of the PBPK model were 1.095 for the tegoprazan and 1.122 for the M1. The evaluation showed that all the developed PBPKs for tegoprazan and M1 described the PK profiles of both tegoprazan and its metabolite, M1, effectively.

### 3.2. Sensitivity Analysis

**Figure 4:**
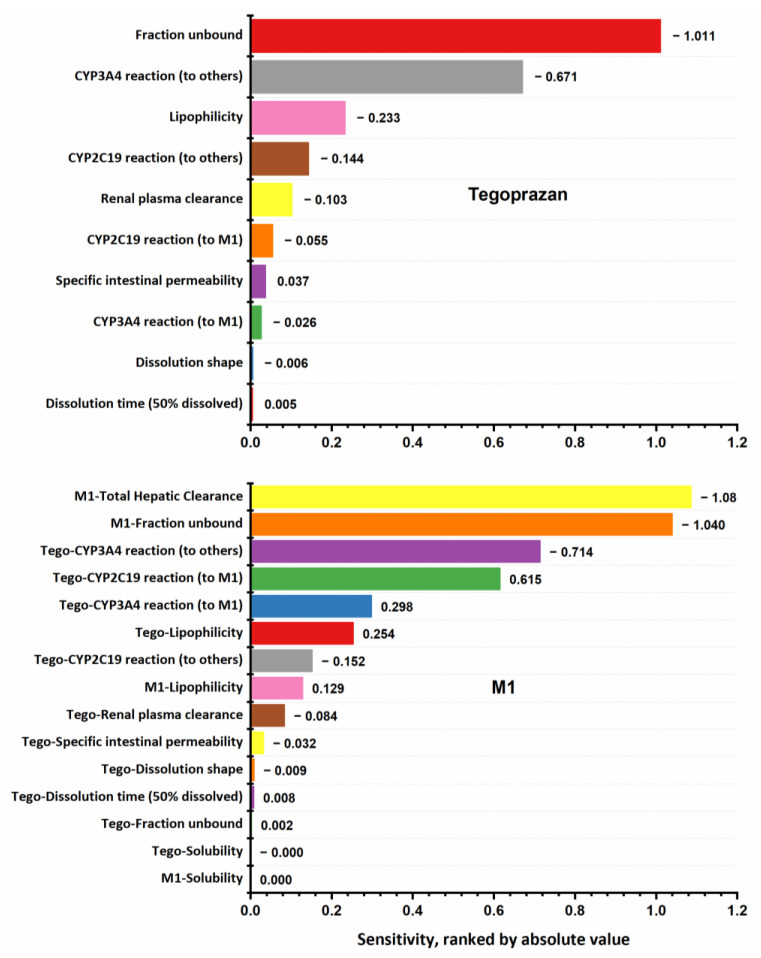
Sensitivity analysis of the developed PBPK model of tegoprazan and M1 to single parameters, calculated as the change in the *AUC*_inf_ of (**upper**) tegoprazan and (**lower**) M1 following a single oral dose of tegoprazan 50 mg under fasted state.

The following parameters were included in the sensitivity analysis: (1) parameters that were optimized, including the lipophilicity (logP) of both tegoprazan and M1; specific intestinal permeability and the clearance of tegoprazan in the presence of rCYPs; and the total hepatic clearance of M1 and (2) parameters that might have significant variation due to calculation methods, including the fraction unbound in plasma and the solubility of tegoprazan and M1. The results of the sensitivity analysis are presented in Figure 4. The analysis revealed that the PK of tegoprazan is mainly sensitive to the fraction unbound in plasma, the intrinsic clearance of tegoprazan via CYP3A4 to form other metabolites, lipophilicity, and the intrinsic clearance of tegoprazan via CYP2C19 to form other metabolites, and renal plasma clearance. The PK of M1 is mainly sensitive to the following parameters: the total hepatic clearance of M1, the fraction unbound of M1, and the hepatic metabolism of tegoprazan.

### 3.3. Prediction DDI Profiles of Tegoprazan

**Figure 5:**
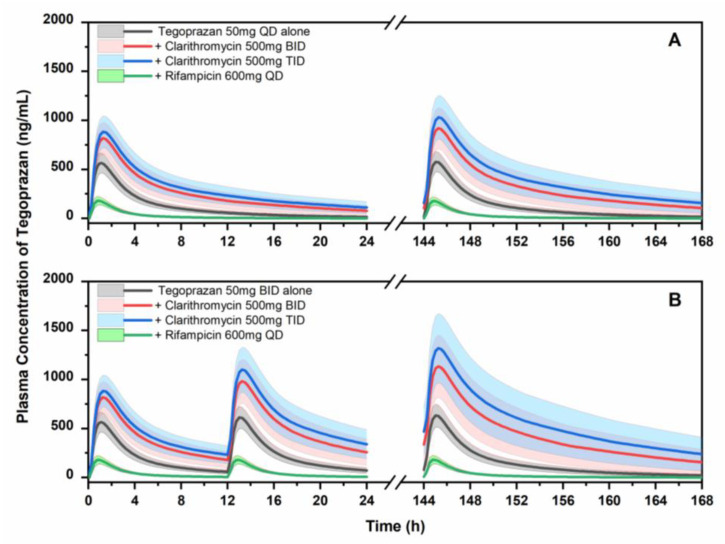
The PK profiles of tegoprazan following the administration of tegoprazan alone or with CYP3A4 perpetrators in a variety of scenarios. (**A**) Tegoprazan, 50 mg, once daily; (**B**) Tegoprazan, 50 mg, twice daily. Dose of clarithromycin: 500 mg, twice or three times daily. Dose of rifampicin: 600 mg, once daily.

As CYP3A4 is the major enzyme accounting for the metabolism of tegoprazan, a DDI between tegoprazan and other CYP3A4 perpetrators is expected. Since the PBPK model for tegoprazan was successfully developed, in this section, we applied the developed model in predicting the DDI profiles of tegoprazan with CYP3A4 perpetrators. Clarithromycin (a strong inhibitor of CYP3A4) and rifampicin (a strong inducer of CYP3A4) were selected as case studies for CYP3A4 perpetrators. The PK profiles of tegoprazan when the drug was administered alone and those when the drug was coadministered with the perpetrators in different scenarios were simulated and compared to each other. The corresponding plasma concentration–time curve and the PK parameters of tegoprazan after the first dose and in the steady state are illustrated in Table 2 and Table 3 and Figure 5. It can be observed that clarithromycin significantly increased tegoprazan exposure. For example, the *AUC*_inf_ and *C*_max_ in the steady state of tegoprazan (50 mg, once daily) increased in magnitude by 3.78-fold (from 2994.4 to 11,312.0 ng × h/mL) and 1.81-fold (from 590.7 to 1066.3 ng/mL), respectively, when the drug was coadministered with clarithromycin (500 mg, twice daily). When the tegoprazan was administered twice daily, the *AUC*_inf_ and *C*_max_ of the tegoprazan increased by 4.54-fold (from 3452.5 to 15,673.1 ng × h/mL) and 2.05-fold (from 649.9 to 1330.9 ng/mL), respectively. Conversely, rifampicin significantly reduced tegoprazan exposure. Through coadministration with rifampicin (600 mg, once daily), the *AUC*_inf_ and *C*_max_ of tegoprazan decreased by 5.26-fold (from 2994.4 to 56.8 ng × h/mL) and 3.28-fold (from 590.7 to 180.1 ng × h/mL), respectively.

## 4. Discussion

The present study reports the successful development of a PBPK model for tegoprazan and the metabolite M1 after administering single or repeated tegoprazan doses, covering an extensive dose range (from 50 to 400 mg) in both the fasted and fed states.

According to previous reports, tegoprazan is primarily metabolized by CYP3A4 and partially by CYP2C19 [4,5,7]. A recent in vitro study by Jeong et al. [4], which studied the stability of tegoprazan in the presence of rCYPs, confirmed the major roles of these two enzymes in the metabolism of tegoprazan. Herein, the intrinsic clearance rates of tegoprazan were estimated as 0.855 and 0.614 µL/min/pmol protein, respectively, in the presence of CYP3A4 and CYP2C19. The intrinsic clearances caused by rCYP2C9, 2C8, 2E1, and 2D6 were comparatively negligible (0.140, 0.060, 0.030, and 0.020 µL/min/pmol protein, respectively). Therefore, in the present study, the metabolism reactions by CYP3A4 and CYP2C19 were included in the PBPK model to model the hepatic clearance of tegoprazan.

First, a PBPK model for only tegoprazan was developed using hepatic clearance (through CYP3A4 and CYP2C19 reactions, regardless of metabolic products) and renal elimination. The total hepatic clearance of tegoprazan caused by each enzyme was estimated. The estimated parameters were then used as initial values to develop a PBPK model for both tegoprazan and M1. The fraction of tegoprazan used to form M1 or other metabolites from the total hepatic clearance was estimated. Accordingly, the PBPK model for tegoprazan and M1 predicted that a sum of 8.3% of the tegoprazan was transformed into M1. Of these, CYP3A4 accounts for the transformation of 2.71%, and CYP2C19 accounts for 5.57% of the administered dose. In addition, 83.5% of the tegoprazan was transformed into metabolites other than M1. Of these, CYP3A4 accounts for the transformation of 68.9%, and CYP2C19 accounts for 14.6% of the administered dose. In summary, 91.8% of the tegoprazan underwent hepatic metabolism, 71.6% via CYP3A4, and 20.2% via CYP2C19. These results are consistent with the published data, in which it was reported that CYP3A4 accounts for approximately 75% of the metabolism of tegoprazan. In addition, around 6.8% of the tegoprazan was transformed into M1 [1,3,18]. The renal plasma clearance (0.297 mL/min/kg, equal to 1.31 L/h) of the tegoprazan was identified from a clinical study [18] without further fitting. Accordingly, our PBPK model predicted that 7.82% of the administered tegoprazan would be excreted via the kidneys. This value was also reasonably close to the published data, wherein a clinical value of 5% was reported [18]. Therefore, the PBPK model developed for tegoprazan in this study can be considered a reasonable model. In addition, based on the sensitivity analysis result, the developed tegoprazan model can be considered certain. In this model, the lipophilicity, specific intestinal permeability, dissolution profile, and hepatic metabolism reactions were parameters obtained with model fitting. Among them, the dissolution profiles and specific intestinal permeability were not the sensitive parameters for the tegoprazan PK profile. The lipophilicity and hepatic reactions were sensitive parameters affecting the PK profile of tegoprazan, and they were estimated based on predicted data from verified software (logP, ALOGPS, and ChemAxon [17]) or experimental data (hepatic reactions [4,12,13]). The fraction unbound and renal plasma clearance were sensitive parameters affecting the PK profile of tegoprazan, and they were taken from the literature without any modifications. Therefore, the sensitivity of the developed PBPK model with the initial input parameters need not be considered.

As M1 is a metabolic product of tegoprazan, the parameter modeling for the absorption phase of M1 is not imperative. Therefore, the solubility and intestinal permeability of M1 were set as the default within PK-Sim^®^. The formation of M1 was modeled using the hepatic reactions of CYP3A4 and CYP2C19. The sensitivity analysis results for the M1 model were that the PK of M1 is mainly affected by the total hepatic clearance, the fraction unbound in the plasma of M1, and the hepatic clearance of tegoprazan. In this study, the fraction unbound in the plasma of M1 was identified based on the literature. The hepatic reactions of tegoprazan to form M1 were optimized based on in vitro and clinical studies. The lipophilicity of M1 was assumed to be the same as that of tegoprazan to ensure the certainty of the parameter estimation for M1. As discussed above, the developed PBPK model of tegoprazan is considered certain. In contrast, for M1, the development of the PBPK model for the compound needed to be considered. The lipophilicity and the total hepatic clearance were estimated with model fitting. The total hepatic clearance was the most sensitive parameter that affected the PK of M1, but there was a lack of experimental data to estimate. Therefore, as both the parameters of logP and the total hepatic clearance of M1 were open to estimation, the parameters could be estimated for non-reasonable values, though the best fit was obtained. For example, the logP of M1 could be estimated at a value significantly higher than the logP of tegoprazan. Therefore, to ensure the estimation of a reasonable logP value and, consequently, a reasonable total hepatic clearance for M1, the logPs of M1 and tegoprazan were simultaneously estimated and forced to receive the same value. This method was performed considering that the logP values for tegoprazan and M1 were not far different. They were predicted to be 2.32 for tegoprazan and 2.10 for M1 (ChemAxon), 2.91 for tegoprazan, and 2.67 for M1 (ALOGPS) [4,17]. Using this method, a value of 1.75 for the logPs of tegoprazan and M1 was obtained. It might be considered reasonable because the values were not too different from the predicted values for both drugs (1.75 vs. 2.32 and 2.91 for tegoprazan, and 1.75 vs. 2.10 and 2.67 for M1).

Food effects were included in the PBPK model for tegoprazan and M1 in this study. In the fed state, the pH in the stomach and the gastric-emptying function increased significantly to 5.5 and then decayed exponentially [26]. In addition, the rate of gastric emptying, which controls the transport of the drug to the absorption sites in the intestine, changed. These changes occurred automatically within PK-Sim^®^ when a meal event was added to the simulation. However, the change in the dissolution profile of the compound from the tablet formulation due to the change in the digestive juice properties and the change in the interface of the tablet with the digestive juice were not simulated. Therefore, the dissolution profile of the tegoprazan formulation under the fed state was estimated separately from that of the fasted state in this study. Accordingly, the developed PBPK model describes the PK of both tegoprazan and M1 under fasted and fed conditions effectively and covers an extensive dose range (from 50 to 400 mg) following a single dose or repeated doses. In total, 100% of the PK parameters (*AUC*_inf_ and *C*_max_) were predicted within the two-fold dimension of the observed values. Approximately 90% of the predicted concentration–time points of the tegoprazan and M1 lay within the two-fold dimension of the observed concentrations. Therefore, our developed PBPK model is reasonable and applicable to the prediction of the PK profiles of tegoprazan and M1 and the prediction of the DDI potential of tegoprazan with interested perpetrators.

This study simulated the DDI potential of tegoprazan and CYP3A4 perpetrators. Clarithromycin, a potent inhibitor of CYP3A4, and rifampicin, a strong inducer of CYP3A4, were selected as case studies. The dose scenarios for tegoprazan, clarithromycin, and rifampicin were selected based on their clinical dosage. The results of our research show that clarithromycin significantly increased the exposure of tegoprazan and M1 in every scenario. It was shown that the *AUC*_inf_ and *C*_max_ of the tegoprazan (50 mg, administered twice daily) in the steady state increased up to 4.54- and 2.05-fold, respectively, when tegoprazan was coadministered with clarithromycin (500 mg, administered three times daily). The rifampicin significantly reduced the tegoprazan and M1 exposure. The *AUC*_inf_ and *C*_max_ of the tegoprazan were reduced by 5.71- and 3.51-fold when tegoprazan was coadministered with rifampicin (600 mg, administered once daily). Due to the high DDI potential, the comedication of tegoprazan with CYP3A4 perpetrators should be controlled. The dose adjustment of tegoprazan in cases of comedication with CYP3A4 perpetrators is suggested to maintain therapeutic effects (when comedications with potent CYP3A4 inducers are used) and safety (when comedications with potent CYP3A4 inhibitors are used). The developed PBPK model also applies to the purposes mentioned above if necessary.

In the clinical studies, after repeated administrations of tegoprazan once daily, the observed Cmax in the steady state decreased significantly. Decreases of approximately 40.2% or 52.9% in the Cmax of tegoprazan were reported after repeated 100 mg or 200 mg tegoprazan doses, respectively [18]. In addition, another study reported a decrease of 29.2% in the Cmax of tegoprazan after repeated 100 mg tegoprazan doses [1]. However, in both these studies, no significant difference in the *AUC*_0–24_ of tegoprazan in the steady state and on the first day was reported; Han et al. [18] reported slight decreases of 9.2% or 6.8% in the *AUC*_0–24_, respectively [18]. Meanwhile, He et al. [1] confirmed that there was no statistically significant difference (*p* > 0.05) in the *AUC*_0–24_ [1]. These results suggested a delay in the rate of the absorption process following repeated tegoprazan doses, which prolonged the time taken to reach peak concentration and decreased the Cmax. However, the decreased absorption rate constant did not affect the total absorbed amount of tegoprazan. This assumption is supported by a study by He et al. [1]. Therein, the absorption rate constants of tegoprazan were reported to be 1.01 and 0.49, respectively, in the first dose and the steady state. Tegoprazan is a weak base (pKa of 5.20) and is classified under class II of the Biopharmaceutical Classification System due to its high permeability and low solubility. Given that tegoprazan is a weak base, its solubility is significantly pH-dependent. For instance, it is 0.7 and 0.02 mg/mL at pH 3 and pH 6.8, respectively [27]. It was predicted to be 223 mg/mL at pH 1 (ChemAxon) and 0.0453 mg/mL at pH 7 (ALOGPS) [4,17]. Therefore, it could be expected that the solubility of tegoprazan in the gastric region is significantly decreased following repeated tegoprazan doses due to the increase in the intragastric pH due to its therapeutic effect. In the case of tegoprazan with limited solubility, the absorption might be strongly affected by a decrease in solubility. The fact that the decreased solubility did not significantly alter the total absorbed amount of tegoprazan might have been due to its high permeability.

In the literature, Yoon et al. [5] and Jeong et al. [4] reported PBPK models for tegoprazan. Both studies used the commercially available software Simcyp Simulator to build the model and generate the PK simulations. In the study by Yoon et al. [5], a PBPK model for tegoprazan was developed using five clinical PK datasets following the administration of a single dose (25, 50, and 100 mg) or multiple doses (50 and 100 mg). However, a PBPK model for the metabolite M1 and the effect of food on the PK of tegoprazan (and, consequently, the PK of its metabolite) was not considered [5]. In the study by Jeong et al. [4], a PBPK model for both tegoprazan and M1 was reported. However, the plasma concentration–time profiles of tegoprazan and M1 from only one clinical study were used for the model’s development. To overcome the limitations of the previously developed models of tegoprazan, in this study, a PBPK for both tegoprazan and M1 was developed using all clinical PK studies of tegoprazan and M1 available in the literature. Accordingly, PK profiles of tegoprazan and M1 from 19 clinical PK datasets that covered an extensive dose range (from 50 to 400 mg) were used after single or repeated doses of tegoprazan. In addition, the PK profiles in both the fasted and fed states were used to model the food effects. In this study, a PBPK model for tegoprazan and M1 was built in PK-Sim^®^ (a free-to-everyone modeling and simulation tool). Although this is a free tool, the PBPK model developed in PK-Sim^®^ showed a similar performance compared to the model developed by Simcyp Simulator (a commercial piece of software). For example, in the case of the DDI profile of tegoprazan with clarithromycin, the model developed in this study predicted the observed PK profiles of tegoprazan well. In cases where tegoprazan was administered with clarithromycin, the predicted/observed ratios of the *C*_max_ and *AUC* of tegoprazan were 0.94 and 1.01, respectively, whereas the corresponding ratios for the model developed by Yoon et al. [5] were 0.99 and 0.93.

The PK profile of M1 following the coadministration of tegoprazan and CYP3A4 perpetrators was not simulated in the present study due to the lack of experimental evidence. As seen in Figure 1 and Figure 2 and Tables S1 and S2, the developed PBPK model for tegoprazan and M1 well-described the PK profiles of both tegoprazan and M1 following the administration of tegoprazan in every study. The model also well-described the tegoprazan PK profile, but not M1, following the coadministration of tegoprazan and clarithromycin, a CYP3A4 inhibitor (clinical dataset ID 09). In detail, the model significantly underpredicted the PK of M1 in dataset ID 09 in comparison with dataset ID 08 (tegoprazan was administered alone). IDs 08 and 09 belong to the same study, which was designed to determine the effect of clarithromycin on the PK of tegoprazan [6]. The relative ratio was significantly reduced from 0.99 to 0.72 for *AUC*_inf_ and from 0.71 to 0.52 for *C*_max_. The underprediction of the model for the PK of M1 suggested the involvement of the CYP3A4 in the metabolism of M1. When coadministered with clarithromycin—in addition to the change in the PK of M1 resulting from the change in the transformation from the parent—a change in M1 PK might also result from the direct CYP3A4 inhibition effect on M1 metabolism. However, the elimination of M1 caused by CYP3A4 was not specifically modeled, and the increase in the PK of M1 due to the inhibition of its elimination was not considered in the developed model due to the lack of experimental evidence. Consequently, the underprediction of the PK of M1 was observed. We suggest that further studies are needed to identify the contribution of CYP3A4 to the PK of M1.

There are some limitations in our study. The change in the PK of tegoprazan (and, consequently, of its metabolite, M1) after repeated tegoprazan doses was not included in the model. Therefore, when performing the simulation, the predicted concentration–time curves of tegoprazan after repeated doses were higher than those of the observed data. Interestingly, while the exposure of tegoprazan was decreased after repeated tegoprazan doses, the exposure of its metabolite, M1, was not. In some studies (for instance, study IDs 08, 09, and 17), the observed exposure of M1 was even higher than the predicted values. Meanwhile, all the PK profiles of M1 at the first dose were well-described. These results suggest that the decrease in the PK of tegoprazan after repeated doses might arise from changes in gastric pH, as well as other physiological or drug-specific factors. The autoinduction in the CYP3A4 and/or CYP2C19 enzymes could be one of the possible explanations for the conflicting change in the exposure of tegoprazan and M1. Further studies need to be conducted to understand tegoprazan and M1 fully. Currently, we are developing a model to connect the PK and pharmacodynamics (PD) of tegoprazan in humans. The therapeutic effect on the pH change of tegoprazan is included in the model. The model is expected to overcome the limitation of the pH effect following repeated doses of tegoprazan observed in this study. In addition, due to a lack of information about the elimination of M1, the liver was assumed to be the major elimination pathway of M1. A parameter of total plasma clearance was used to cover all the possible hepatic metabolic pathways. The contribution of the CYP3A4 enzyme was not specifically included in the model of M1; therefore, the PK profile of M1 following the administration of tegoprazan with CYP3A4 perpetrators could not be performed.

## 5. Conclusions

A PBPK model for both tegoprazan and the major active metabolite M1 was developed in this study to overcome the limitations of previously published models [4,5]. All clinical PK studies of tegoprazan and M1 available in the literature were used for model development. The effects of food on the PKs of tegoprazan were included. Although the model was built using a free tool, PK-Sim^®^, a similar performance compared with the model developed by Simcyp Simulator was observed. With this developed PBPK model, lots of drug interactions commonly coadministered with tegoprazan could be evaluated. This developed model was then applied to the prediction of the DDI profiles of tegoprazan with CYP3A4 perpetrators. Our results showed that clarithromycin significantly increased tegoprazan exposure. Rifampicin significantly reduced tegoprazan exposure. Since the DDI was significant, the comedication of tegoprazan with these CYP3A4 perpetrators should be controlled. In addition, the dose adjustment for tegoprazan in cases of comedication with CYP3A4 perpetrators is suggested to maintain therapeutic effects (when comedications with potent CYP3A4 inducers are used) and maintain safety (when co-medications with potent CYP3A4 inhibitors are used). The developed PBPK model could also be applied to the purposes mentioned above if necessary. Other than with clarithromycin and rifampicin, using the developed PK model, the DDI profile of tegoprazan with other CYP3A4s can be easily predicted.

## Figures and Tables

**Table 1:** Input parameters and optimized parameters for the PBPK model of tegoprazan and the metabolite M1.

Parameter	Final Value	Unit	Reference Value	Source of Reference
Tegoprazan				
physicochemical properties				
Molecular weight	387.38	g/mol	387.38	[16,17]
Lipophilicity (LogP)	1.75	-	2.32 predicted by ChemAxon 2.91 predicted by ALOGPS	[17]
Fraction unbound in plasma	8.7	%	8.7; 12.4	[2]
Solubility	45.3	mg/L	45.3 predicted by ALOGPS	[17]
Compound type/pKa Strongest basic Strongest acidic	5.20 12.0		5.20/6.07 12.0/11.37	[5,17]
ADME parameters				
Absorption				
Specific intestinal permeability	$1.16\times 10^{-6}$	cm/s	$1.24\times 10^{-3}$ (Peff) $6.84\times 10^{-5}$ (PAMPA Caco-2cell)	[5]
Distribution				
Partition coefficients	PK-Sim^®^ Standards			
Cellular permeabilities	PK-Sim^®^ Standards			
Specific organ permeability	$1.15\times 10^{-4}$	cm/s	$1.15\times 10^{-4}$	PK-Sim^®^
Metabolism Intrinsic metabolic rate in the presence of CYPs				
CYP3A4 to M1	$9.29\times 10^{-3}$	μL/min/pmol recombinant enzyme	0.134	[4]
CYP3A4 to other metabolites	0.236			
CYP2C19 to M1	0.0921		0.154	
CYP2C19 to other metabolites	0.242			
Transport and excretion				
Renal plasma clearance	0.297	mL/min/kg	0.297	[18]
Dissolution profile: Dissolution time and shape	Fasted: 0.942 h and 0.990 Fed: 4.23 h and 0.502			
M1				
physicochemical properties				
Molecular weight	373.40			[4,7]
Lipophilicity (LogP)	1.75		2.10 predicted by ChemAxon 2.67 predicted by ALOGPS	[4]
Fraction unbound in plasma	1	%	1	[2]
Solubility	1.00	ng/mL		assumed
Compound type/pKa (Strongest base)	5.35			[4]
ADME parameters				
Metabolism Total hepatic clearance	0.140	mL/min/kg		

Peff—effective human jejunal permeability; PAMPA—parallel artificial membrane permeability assay; rCYPs—recombinant cytochrome P450s; ADME—absorption, distribution, metabolism, and excretion.

**Table 2:** Predicted PK parameters of tegoprazan following oral administration of tegoprazan (50 mg, QD) with CYP3A4 perpetrators.

Parameters	Unit	Tegoprazan Alone	Tegoprazan with Clarithromycin 500 mg BID		Tegoprazan with Clarithromycin 500 mg TID		Tegoprazan with Rifampicin 600 mg QD	
		Value (Min–Max)	Value (Min–Max)	Fold Increase	Value (Min–Max)	Fold Increase	Value (Min–Max)	Fold Decrease
*AUC*_first_	ng × h/mL	2895.5	7059.3	2.44	9092.3	3.14	578.2	5.01
		1949.1–4155.1	3523.5–11,510.1		4708.8–14,461.4		398.5–845	
*AUC*_SS_	ng × h/mL	2994.4	8198.7	2.74	11312.0	3.78	568.8	5.26
		2008.1–4536	3782.8–16,814.2		5213–21,020.6		392.8–804.6	
*C*_max_first_	ng/mL	576.2	826.0	1.43	893.0	1.55	183.7	3.14
		426.1–740.3	570–1101.3		627.7–1161.1		127.7–254.8	
*C*_max_SS_	ng/mL	590.7	948.2	1.61	1066.3	1.81	180.1	3.28
		428.8–763.3	616.4–1253.4		711.3–1434.6		125.2–248.4	

QD, once daily; BID, twice daily; TID, three times daily; *AUC*, area under the concentration–time curve; *C*_max_, the maximum concentration; the subscript “first”, the first dose; the subscript “SS”, the steady state.

**Table 3:** Predicted PK parameters of tegoprazan following oral administration of tegoprazan (50 mg, BID) with CYP3A4 perpetrators.

Parameters	Unit	Tegoprazan Alone	Tegoprazan with Clarithromycin 500 mg BID		Tegoprazan with Clarithromycin 500 mg TID		Tegoprazan with Rifampicin 600 mg QD	
		Value (Min–Max)	Value (Min–Max)	Fold Increase	Value (Min–Max)	Fold Increase	Value (Min–Max)	Fold Increase
*AUC*_first_	ng × h/mL	2866.9	6680.3	2.33	8900.1	3.10	575.6	4.98
		1917.8–4157.4	3391.8–11,486.7		4638.9–15,811		397.6–845	
*AUC*_SS_	ng × h/mL	3452.5	10984.0	3.18	15673.1	4.54	605.1	5.71
		2235.7–5593.3	4653.1–27,087.4		6398.6–34,092.7		425.6–804.6	
*C*_max_first_	ng/mL	576.2	826.0	1.43	893.0	1.55	183.7	3.14
		426.1–740.3	570–1101.3		627.7–1161.1		127.7–254.8	
*C*_max_SS_	ng/mL	649.9	1163.9	1.79	1330.9	2.05	185.4	3.51
		466.8–848.1	700.9–1625.3		851.9–1892.6		129–248.4	

QD, once daily; BID, twice daily; TID, three times daily; *AUC*, area under the concentration–time curve; *C*_max_, the maximum concentration; the subscript “first”, the first dose; the subscript “SS”, the steady state.

## Data Availability

Not applicable.

## Supplementary Materials

The following supporting information can be downloaded at https://www.mdpi.com/article/10.3390/pharmaceutics15010182/s1: Tables S1 and S2: Clinical studies for PBPK model development; PK-Sim Model File: PBPK model for tegoprazan and M1. Refs. [1,6,7,18,28,29,30] has been cited in supplementary file.
