# Supplementary material for: Development of a Physiologically Based Pharmacokinetic Model for Tegoprazan: Application for the Prediction of Drug–Drug Interactions with CYP3A4 Perpetrators
Source: Pharmaceutics. 2023 Jan 4;15(1):182. doi: 10.3390/pharmaceutics15010182 (PMC9862396; doi:10.3390/pharmaceutics15010182)
Supplement: Supplementary file 1 [file pharmaceutics-15-00182-s001.zip › pharmaceutics-2091469-supplementary/Supplementary Materials/Tables S1 and S2..pdf]

Development of a Physiologically Based Pharmacokinetic Model for Tegoprazan: Application for the Prediction of Drug–Drug Interactions with CYP3A4 Perpetrators

Lien Thi Ngo †, Jaeyeon Lee †, Hwi-yeol Yun * and Jung-woo Chae *

College of Pharmacy, Chungnam National University, Daejeon 34134, Republic of Korea; lienngovn@cnu.ac.kr (L.T.N.); jaeyeon.lee@o.cnu.ac.kr (J.L.)

* Correspondence: hyyun@cnu.ac.kr (H.-y.Y.); jwchae@cnu.ac.kr (J.-w.C.)

† These authors contributed equally to this work as the first authors.

Table S1 Observed and Predicted pharmacokinetics parameters of **tegoprazan** following the administration of tegoprazan in humans

**Training dataset**		***AUC***$_{inf}$ **(ng×h/mL)**			***C***$_{max}$ **(ng/mL)**			**References**	**Description**
Study ID	Dose (mg)	Observed	Predicted	Ratio	Observed	Predicted	Ratio		
ID 01	50	2107.1	2843.5	1.35	567.4	523.3	0.92	Han et al. 2017 [1]	Single dose
ID 04	400	26288.3	23049.4	0.88	4854.1	3657.6	0.75	Han et al. 2017 [1]	Single dose
ID 05	100	6763.6	5951.7	0.88	1380.4	1089.5	0.79	Han et al. 2017 [1]	Multiple, first dose
ID 05	100	4148.1	6145.7	1.48	705.0	1124.9	1.60	Han et al. 2017 [1]	Multiple, last dose
ID 07	100	6223.4	6053.2	0.97	1227.0	1094.9	0.89	Hwang et al. [2]	Single dose
ID 08	100	7737.2	7672.1	0.99	976.8	1212.9	1.24	Ghim et al. 2021 [3]	Multiple doses, tegoprazan alone
ID 09	100	23755.1	23909.4	1.01	2256.1	2113.4	0.94	Ghim et al. 2021 [3]	Multiple doses, tegoprazan with clarithromycin
ID 12	200	11109.3	12445.1	1.12	2535.6	2612.9	1.03	He et al. 2021 [4]	Single dose
ID 14	50	3813.8	2919.4	0.77	635.2	537.4	0.85	Yoon et al. 2021 [5]	Single dose
ID 17	50	3693.6	3218.9	0.87	538.7	566.8	1.05	Jeon et al. 2021 [6]	Multiple doses
ID 19	200	7516.5	8725.3	1.16	910.5	644.6	0.71	Han et al. 2021 [7]	Single dose, fed

**Test dataset**		***AUC***$_{inf}$ **(ng×h/mL)**			***C***$_{max}$ **(ng/mL)**			**References**	**Description**
Study ID	Dose (mg)	Observed	Predicted	Ratio	Observed	Predicted	Ratio		
ID 02	100	5651.4	5903.1	1.04	1227.4	1046.5	0.85	Han et al. 2017 [1]	Single dose
ID 03	200	10358.2	11851.6	1.14	2480.2	2122.5	0.86	Han et al. 2017 [1]	Single dose
ID 06	200	10325.5	11947.1	1.16	2789.2	2235.0	0.80	Han et al. 2017 [1]	Multiple, first dose
ID 06	200	10244.6	12717.2	1.24	1150.4	2303.3	2.00	Han et al. 2017 [1]	Multiple, last dose
ID 10	50	3152.1	3061.5	0.97	715.6	615.2	0.86	He et al. 2021 [4]	Single dose
ID 11	100	5709.6	6093.2	1.07	1275.6	1217.5	0.95	He et al. 2021 [4]	Single dose
ID 13	100	6943.5	6409.9	0.92	978.5	1248.0	1.28	He et al. 2021 [4]	Multiple doses
ID 15	50	3417.9	2919.4	0.85	670.3	537.4	0.80	Yoon et al. 2021 [5]	Single dose, fasted
ID 16	50	3629.1	2896.9	0.80	327.4	331.7	1.01	Yoon et al. 2021 [5]	Single dose, fed
ID 18	200	8455.4	11673.9	1.38	1898.7	2122.5	1.12	Han et al. 2021 [7]	Single dose, fasted

AUC$_{inf}$, area under the concentration-time curve; C_{max}, maximum concentration

Table S2 Observed and Predicted pharmacokinetics parameters of **the metabolite M1** following the administration of tegoprazan in humans

**Training dataset**		***AUC*$_{inf}$ (ng×h/mL)**			**C_{max} (ng/mL)**			**References**	**Description**
Study ID	Dose (mg)#	Observed	Predicted	Ratio	Observed	Predicted	Ratio		
ID 07	100	9058.2	9642.8	1.06	395.6	307.6	0.78	Hwang et al. [2]	Single dose
ID 08	100	23865.9	23521.0	0.99	1211.2	855.4	0.71	Ghim et al. 2021 [3]	Multiple doses, tegoprazan alone
ID 09¥	100	66841.0	47842.4	0.72	2696.4	1407.9	0.52	Ghim et al. 2021 [3]	Multiple doses, tegoprazan with clarithromycin
ID 12	200	16838.8	19513.8	1.16	958.1	731.1	0.76	He et al. 2021 [4]	Single dose
ID 14	50	5230.4	4677.0	0.89	183.5	146.2	0.80	Yoon et al. 2021 [5]	Single dose
ID 17	50	4621.8	4700.5	1.02	135.9	145.5	1.07	Jeon et al. 2021 [6]	Multiple doses
ID 19	200	9222.7	12210.753	1.32	303.1	367.5	1.21	Han et al. 2021 [7]	Single dose, fed
**Test dataset**		***AUC*$_{inf}$ (ng×h/mL)**			**C_{max} (ng/mL)**			**References**	**Description**
Study ID	Dose (mg)#	Observed	Predicted	Ratio	Observed	Predicted	Ratio		
ID 10	50	4430.7	4832.7	1.09	243.5	185.1	0.76	He et al. 2021 [4]	Single dose
ID 11	100	7678.0	9680.7	1.26	493.2	360.1	0.73	He et al. 2021 [4]	Single dose
ID 13	100	12013.9	13693.9	1.14	547.8	551.4	1.01	He et al. 2021 [4]	Multiple doses
ID 15	50	4317.6	4677.0	1.08	144.0	146.2	1.02	Yoon et al. 2021 [5]	Single dose, fasted
ID 16	50	4621.8	4700.5	1.02	135.9	145.5	1.07	Yoon et al. 2021 [5]	Single dose, fed
ID 18	200	11692.8	18847.8	1.61	586.0	569.5	0.97	Han et al. 2021 [7]	Single dose, fasted

AUC$_{inf}$, area under the concentration-time curve; C_{max}, maximum concentration

Dose of tegoprazan; ¥this dataset was not used for the model development.

References

1. Han, S.; Choi, H.Y.; Kim, Y.H.; Nam, J.Y.; Kim, B.; Song, G.S.; Lim, H.-S.; Bae, K.-S. Randomised clinical trial: safety, tolerability, pharmacokinetics, and pharmacodynamics of single and multiple oral doses of tegoprazan (CJ-12420), a novel potassium-competitive acid blocker, in healthy male subjects. *Aliment. Pharmacol. Ther.* **2019**, *50*, 751–759, doi:10.1111/apt.15438.
2. Hwang, J.G.; Yoo, H.; Lee, J.W.; Song, G.S.; Lee, S.; Kim, M.-G. Comparison of pharmacokinetic characteristics of two Tegoprazan (CJ-12420) formulations in healthy male subjects. *Transl. Clin. Pharmacol.* **2019**, *27*, 80, doi:10.12793/tcp.2019.27.2.80.
3. Ghim, J.; Chin, M.C.; Jung, J.; Lee, J.; Kim, S.; Kim, B.; Song, G.S.; Choi, Y.; Shin, J. Pharmacokinetics and Pharmacodynamics of Tegoprazan Coadministered With Amoxicillin and Clarithromycin in Healthy Subjects. *J. Clin. Pharmacol.* **2021**, *61*, 913–922, doi:10.1002/jcph.1805.
4. He, J.; Cao, G.; Yu, J.; Wang, J.; Cheng, N.; Wu, J.; Zhang, J.; Wu, X.; Zhang, B.; Lu, J.; et al. Safety, Tolerability and Pharmacokinetics of Single Ascending and Multiple Oral Doses of Tegoprazan in Healthy Chinese Subjects. *Clin. Drug Investig.* **2021**, *41*, 89–97, doi:10.1007/s40261-020-00986-4.
5. Yoon, D.Y.; Sunwoo, J.; Shin, N.; Kim, A.R.; Kim, B.; Song, G.S.; Jang, I.; Lee, S. Effect of meal timing on pharmacokinetics and pharmacodynamics of tegoprazan in healthy male volunteers. *Clin. Transl. Sci.* **2021**, *14*, 934–941, doi:10.1111/cts.12958.
6. Jeon, J.-Y.; Kim, S.-Y.; Moon, S.J.; Oh, K.; Lee, J.; Kim, B.; Song, G.S.; Kim, M.-G. Pharmacokinetic Interactions between Tegoprazan and Metronidazole/Tetracycline/Bismuth and Safety Assessment in Healthy Korean Male Subjects. *Clin. Ther.* **2021**, *43*, 722–734, doi:10.1016/j.clinthera.2021.01.026.
7. Han, S.; Choi, H.Y.; Kim, Y.H.; Nam, J.Y.; Kim, B.; Song, G.S.; Lim, H.-S.; Bae, K.-S. Effect of Food on the Pharmacokinetics and Pharmacodynamics of a Single Oral Dose of Tegoprazan. *Clin. Ther.* **2021**, *43*, 1371–1380, doi:10.1016/j.clinthera.2021.06.007.
